# Dynamic Analysis of Stool Microbiota of Simmental Calves and Effects of Diarrhea on Their Gut Microbiota

**DOI:** 10.3390/biology13070520

**Published:** 2024-07-13

**Authors:** Qianxun Wang, Mula Na, Shiyu Jia, Miao Sun, Song Gao, Shiwei Pan, Wu Dong, Yang Song, Jingfeng Yang

**Affiliations:** College of Animal Science and Technology, Inner Mongolia Minzu University, Tongliao 028000, China; jlwangqianxun@163.com (Q.W.); 15148192790@163.com (M.N.); 18347589300@163.com (S.J.); 15947674804@163.com (M.S.); 15114739188@163.com (S.G.); psw52122@163.com (S.P.); dongwu@imun.edu.cn (W.D.)

**Keywords:** calf diarrhea, intestinal flora, high-throughput 16S rRNA

## Abstract

**Simple Summary:**

Simmental cattle is a renowned breed that is extensively raised because of its high beef and milk fat content. The early establishment of the gut microbiota is closely related to the gut health of calves. However, the structure and function of the gut microbiota in Simmental calves before weaning remain unclear. The development of gut microbiota in neonatal calves is a multifaceted process affected by numerous factors. Gastrointestinal microbiota profoundly affect animal health, productivity, and disease susceptibility, necessitating further study of specific microbial functions during early development. Diarrhea stands out as the most prevalent ailment among lactating calves. Changes in the structure and functionality of the gut microbiota can precipitate gut disorders, ultimately leading to diarrhea. Therefore, thorough investigations of the early colonization patterns of Simmental calves, along with an understanding of the gut microbiota in healthy calves versus those afflicted by diarrhea, have significant implications for diarrhea prevention and development of probiotic interventions.

**Abstract:**

The objective of this study was to explore the dynamic changes in the gut microbiota of Simmental calves before weaning and to compare the microbial composition and functionality between healthy calves and those with diarrhea. Fourteen neonatal Simmental calves were divided into a healthy group (*n* = 8) and a diarrhea group (*n* = 6). Rectal stool samples were collected from each calf on days 1, 3, 5, 7, 9, 12, 15, 18, 22, 26, 30, 35, and 40. High-throughput sequencing of the 16S rRNA gene V1–V9 region was conducted to examine changes in the gut microbiota over time in both groups and to assess the influence of diarrhea on microbiota structure and function. *Escherichia coli*, *Bacteroides fragilis*, and *B. vulgatus* were the top three bacterial species in preweaning Simmental calves. Meanwhile, the major functions of the fecal microbiota included “metabolic pathways”, “biosynthesis of secondary metabolites”, “biosynthesis of antibiotics”, “microbial metabolism in diverse environments”, and “biosynthesis of amino acids”. For calves in the healthy group, PCoA revealed that the bacterial profiles on days 1, 3, 5, 7, and 9 differed from those on days 15, 18, 22, 26, 30, 35, and 40. The profiles on day 12 clustered with both groups, indicating that microbial structure changes increased with age. When comparing the relative abundance of bacteria between healthy and diarrheic calves, the beneficial *Lactobacillus johnsonii*, *Faecalibacterium prausnitzii*, and *Limosilactobacillus* were significantly more abundant in the healthy group than those in the diarrhea group (*p* < 0.05). This study provides fundamental insights into the gut microbiota composition of Simmental calves before weaning, potentially facilitating early interventions for calf diarrhea and probiotic development.

## 1. Introduction

Simmental cattle, a renowned dual-purpose breed originating from Switzerland, are noted for their exceptional performance in both meat and milk production [1]. Consequently, Simmental cattle are primarily utilized in China for cross-breeding with dairy and beef cattle [2]. Inner Mongolia is the main area for beef cattle breeding in China, with Tongliao serving as the core area. In Tongliao, the beef cattle population exceeds three million, with Simmental cattle being the main breed. Calf health is the most critical aspect of beef cattle breeding [3]. Currently, calf diarrhea causes the highest rates of morbidity and mortality, resulting in significant economic losses [4]. Limited information is available on the fecal microbiota of bovines under extensive grazing conditions. Therefore, from economic, ecological, and health perspectives, it is crucial to assess the bacterial diversity (from phylum to species) in the intestines of domestic ruminants.

Early colonization of the gut microbiota significantly affects calf gut health [5]. For neonatal calves, microbial colonization is a complex and dynamic process [6], influenced by host–microbial interactions and various external factors [7], such as maternal microbiota, birth process, diet, antibiotics, and weaning status. Studies indicate that the intestinal flora of ruminants begins to develop during the fetal period, with detectable microflora appearing in the rumen, cecum, meconium, and even amniotic fluid of calves after approximately 5 months of pregnancy [8,9]. After birth, the intestinal microbiota of neonatal calves undergo rapid changes. Within approximately 8 h, *Escherichia coli* and *Streptococcus* colonize all gastrointestinal regions, followed by lactic acid bacteria and *Clostridium perfringens* [10]. Lactic acid bacteria dominate both the cecum and stool samples from the second day to one week after birth [11]. In 3-week-old calves, dominant bacterial genera include *Bacteroides*, *Prevotella*, *Coccus*-*Useriella*, and *Faebacillus* [12], followed by the appearance of *Lactococcus flavus* and cellulolytic bacteria by the fifth week [11]. By 12 weeks, *Prevotella*, *Bacteroides*, *Clostridium*, and *Eubacterium* are the main intestinal microbiota in calves [13]. Although these dynamic microbial changes have been extensively studied in Holstein calves, research on Simmental calves exploring microbial colonization and function remains limited [14].

Calf diarrhea stands as a leading cause of mortality before weaning, with approximately 53.4% of calf deaths in South Korea attributed to this condition [15]. A 2018 NAHMS study reported that 39% of calf deaths within the first 3 weeks of life in the United States were due to diarrhea [16]. Recent studies have shown mortality rates of 7.6% in Canada and 5.3% in Belgium [17,18]. Despite a decline in the mortality rate of US dairy calves from 11% in 2007 to 5% in 2014, the overall incidence remains high [19]. The factors contributing to neonatal calf diarrhea are multifaceted, primarily involving nutritional factors, intestinal inflammation, stress, and pathogenic infections [20]. Antibiotics, such as β-lactams and sulfonamides, are commonly used to treat calf diarrhea. Overuse of antibiotics can disrupt the gut microbiota of calves, leading to intestinal disorders. Specifically, antibiotics such as methylene salicylic acid and bacitracin, used in calf diarrhea treatment, may enhance the colonization of potential pathogens such as *E. coli*, *Enterococcus*, and *Shigella* in calf intestines, thereby affecting the intestinal microbial balance [21].

Meanwhile, the use of antibiotics also affects the metabolic patterns of intestinal microbiota and nutrient absorption, potentially promoting colonization by drug-resistant bacteria and increasing the risk of infection in preweaning calves [22]. Additionally, antibiotic residues can adversely affect the environment, underscoring the importance of exploring alternative antibiotics for the prevention and treatment of calf diarrhea. Probiotics, including *Bifidobacterium pseudocatenulatum*, *Lactobacillus acidophilus* [23], and *Bacillus subtilis* [24,25], can serve as sustainable options for the prevention and treatment of diarrhea in young calves. They can resist pathogen adhesion and enhance the intestinal barrier function, thereby reducing intestinal damage. Studies have shown that *Bifidobacterium pseudomidobacterium* produces lactic acid and short-chain fatty acids (SCFAs) to facilitate the prevention and treatment of calf diarrhea [26]. Moreover, the proliferation of probiotics in the intestine can lower undigested carbohydrate levels, thus reducing the risk of diarrhea resulting from osmotic gradient disruption [27]. The above findings were mainly based on Holstein calves, and there is a dearth of reports on probiotic applications and diarrhea treatment in Simmental calves.

This study investigated changes in the gut microbiota of healthy Simmental calves and those who experienced diarrhea before weaning over time and predicted intestinal microbial functions. Additionally, we compared the microbial composition and functions of calves in the health and diarrhea groups before, during, and after the diarrhea period.

## 2. Materials and Methods

### 2.1. Animal Breeding

The experiment was conducted at a commercial ranch in Tongliao City, Inner Mongolia, from February to April 2023, following protocols approved by the Laboratory Animal Welfare and Ethics Committee of the College of Animal Science and Technology, Inner Mongolia Minzu University (protocol code: 2022058). In total, 14 neonatal Simmental calves (7 males and 7 females, weighing 39 ± 5 kg) were randomly selected upon birth and enrolled in the study. They were managed according to the standard feeding protocol of the ranch. Immediately after birth, all calves were separated from their maternal cows and housed in individual hutches after umbilical cord disinfection. Each calf was housed in a calf island (1.8 m × 1.4 m × 1.2 m), with bedding changed every 7 days to maintain a healthy environment. Within 1 h of birth, each calf received 4 L of colostrum, followed by an additional 2 L within 8–10 h. Subsequently, they were fed 5 L of whole milk per day. The calves had ad libitum access to calf starter on the 3rd day after birth.

### 2.2. Daily Health Monitoring

Calves were monitored and assessed daily before morning and evening feeding. Parameters, including nasal and eye discharge, coughing, umbilical cord inflammation, rectal temperature, dehydration status, and overall condition, were recorded using the specific scoring criteria outlined in a previously published article [28]. Fecal samples were collected and scored based on fecal fluidity: 1 = normal, 2 = soft, 3 = runny, or 4 = watery. Calves with a fecal score of 3 or 4 were classified as diarrheic [29]. The detailed scoring criteria are provided in Supplementary File S1.

### 2.3. Grouping

The calves were categorized into two groups according to the occurrence of diarrhea during the study period: diarrheal group (A) (*n* = 6) and healthy group (B) (*n* = 8). All calves in the diarrheal group experienced diarrhea starting on day 5 and recovered by day 9 after treatment with an oral electrolyte solution. Calves in the healthy group remained healthy throughout the experimental period. Stool samples were collected from all calves at 8:00 am on days 1, 3, 5, 7, 9, 12, 15, 18, 22, 26, 30, 35, and 40 after birth. Fecal samples were temporarily stored in liquid nitrogen and subsequently transferred to a −80 °C refrigerator for long-term storage.

### 2.4. High-Throughput 16S Ribosomal RNA Gene Sequencing

Total genomic DNA was extracted from stool samples using the TGuide S96 Magnetic Stool DNA Kit (Tiangen Biotech, Beijing, China), following the manufacturer’s instructions. The quality and quantity of the extracted DNA were assessed by electrophoresis on a 1.8% agarose gel, and the DNA concentration and purity were determined using a NanoDrop 2000 UV-Vis spectrophotometer (Thermo Scientific, Wilmington, DE, USA). The full-length 16S rRNA gene was amplified using primer pair 27F: AGRGTTTGATYNTGGCTCAG and 1492R: TASGGHTACCTTGTTASGACTT. Both the forward and reverse 16S primers were tagged with sample-specific PacBio barcode sequences to enable multiplexed sequencing. Barcoded primers were selected to minimize chimera formation compared with an alternative protocol involving a second PCR reaction. PCR amplification was performed using the KOD One PCR Master Mix (Toyobo Life Science, Wuhan, China) for 25 cycles, comprising an initial denaturation at 95 °C for 2 min, followed by 10 s of denaturation at 98 °C, annealing at 55 °C for 30 s, extension at 72 °C for 1 min 30 s per cycle, and a final step at 72 °C for 2 min. The total PCR amplicons were purified using VAHTSTM DNA Clean Beads (Vazyme, Nanjing, China) and quantified with the Qubit dsDNA HS Assay Kit and Qubit 3.0 Fluorometer (Invitrogen, Thermo Fisher Scientific, Hillsboro, OR, USA). After individual quantification, amplicons were pooled in equal proportions. SMRTbell libraries were then generated from the amplified DNA using the SMRTbell Express Template Prep Kit 2.0 (Beijing, China) according to the manufacturer’s instructions (Pacific Biosciences, Beijing, China). The purified SMRTbell libraries derived from the pooled and barcoded samples were sequenced on a PacBio Sequel II platform (Beijing Biomarker Technologies Co., Ltd., Beijing, China) using Sequel II binding kit 2.0 (Beijing, China).

### 2.5. Bioinformatic Analysis

Qualified sequences exceeding a 97% similarity threshold were assigned to operational taxonomic units (OTUs) using USEARCH (version 10.0). Taxonomic annotation of the OTUs was conducted using the Naive Bayes classifier in QIIME2 [30], utilizing the SILVA database [31] (release 138.1) with a confidence threshold of 70%. The α-diversity test was performed to determine the complexity of species diversity in each sample using QIIME2 (version 2020.6) software. Beta diversity was analyzed using principal co-ordinate analysis (PCoA) to evaluate species diversity across the samples. Bacterial abundance and diversity were compared using one-way analysis of variance. The gut microbiome was considered significant if its relative abundance exceeded 0.1% and was present in over half of the animals in at least one age group. FAPROTAX software (https://anaconda.org/bioconda/fastx_toolkit, accessed on 25 May 2024) was employed to predict the function of intestinal fecal microorganisms in calves, focusing on metabolic pathways with a CPM > 5 observed in at least 50% of the animals within each treatment group.

### 2.6. Statistical Analysis

Data were analyzed using Microsoft Excel 2019 and SPSS 25.0. A nonparametric Wilcoxon test was performed to assess differences in the α-diversity index and relative abundance of microbiota between the control and diarrhea groups on the same sampling day. The top five bacterial species were compared between adjacent time points within each group, with *p* < 0.05 indicating statistical significance and 0.05 < *p* < 0.10 suggesting a trend towards significance. In addition, a similarity analysis between the two groups was conducted using ANOSIM. The results were interpreted as follows. When *p* < 0.05 and *r* < 0.5, the intestinal microbiota between the two groups were similar; when *p* < 0.05 and *r* > 0.75, the microbiota were completely different; when 0.5 < *r* < 0.75, they were different; and 0.3 < *r* < 0.5 indicated a tendency towards difference. No significant differences were observed for *r* < 0.3. A Wilcoxon test was applied to compare intergroup differences between the control and diarrhea groups on the same sampling day for gut microbiota function [32], with *p* < 0.05 indicating statistical significance.

### 2.7. Nucleotide Sequence Accession Numbers

All sequences were deposited in the NCBI Sequence Read Archive and are publicly accessible under accession number PRJNA1072024. These files can be accessed via the following link: https://www.ncbi.nlm.nih.gov/bioproject/PRJNA1072024 (accessed on 1 February 2024).

## 3. Results

### 3.1. Data Collection and Diversity of Gut Microbiota

**Figure 1:**
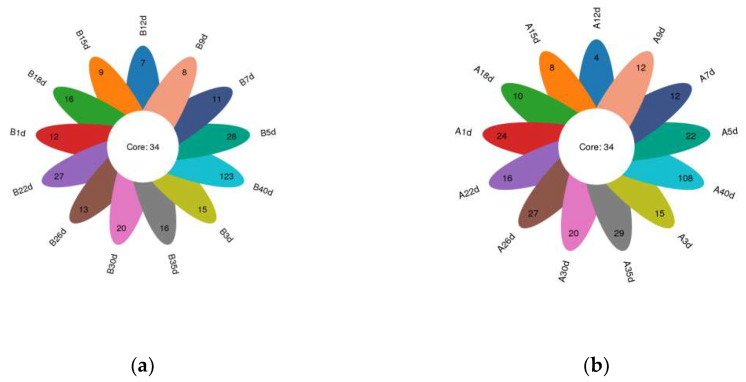
Petalograms of intestinal microbial OTUs at different time points during the preweaning period in Simmental calves. (**a**) Healthy group and (**b**) diarrheal group.

In total, amplicon sequencing of preweaning Simmental calves’ intestinal microbiota yielded 2,185,694 high-quality sequences, which were assigned to a total of 1122 OTUs based on 97% nucleotide sequence similarity (Supplementary File S2). Both the sparsity and rank abundance curves exhibited saturation trends, indicating that the depth and uniformity of sequencing met the requirements for subsequent analyses (Supplementary File S3). The calves in the healthy group exhibited 34 shared OTUs across various time points, with additional 12, 15, 28, 11, 8, 7, 9, 16, 27, 13, 20, 16, and 123 OTUs specific to days 1, 3, 5, 7, 9, 12, 15, 18, 22, 26, 30, 35, and 40 (Figure 1a). Similarly, calves in the diarrheal group displayed 34 shared OTUs across different time points, with additional 24, 15, 22, 12, 12, 4, 8, 10, 16, 27, 20, 29, and 108 OTUs specific to days 1, 3, 5, 7, 9, 12, 15, 18, 22, 26, 30, 35, and 40 (Figure 1b). Additionally, α-diversity indices (ACE, Chao1, PD-whole-tree, Shannon, and Simpson) were compared at adjacent time points for calves in both healthy and diarrheal groups, revealing no significant differences throughout the preweaning period (Supplementary File S3).

**Figure 2:**
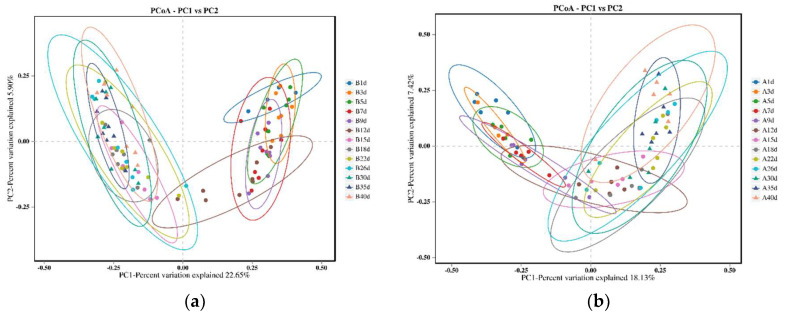
Comparison of bacterial profiles of calves across different sampling points using principal co-ordinate analysis (PCoA). (**a**) PCoA plot generated using unweighted Jaccard for 13 different time points of calves in the healthy groups. The two principal components explained 22.65% and 5.90% of the variance. (**b**) PCoA plot generated using unweighted Jaccard for 13 different time points of calves in the diarrheal groups. The two principal components explained 18.13% and 7.42% of the variance.

The PCoA results revealed that the microbial profiles of calves in the healthy group exhibited similar structures on days 1, 3, 5, 7, and 9, as well as on days 15, 18, 22, 26, 30, 35, and 40. However, on day 12, the microbial profiles of the calves intersected with two distinct clusters (Figure 2a). Similarly, for calves in the diarrheal group, the microbial profiles were similar on days 1, 3, and 5, as well as on days 15, 18, 22, 26, 30, 35, and 40. However, on days 7, 9, and 12, the microbial profiles intersected with the aforementioned two clusters (Figure 2b).

### 3.2. Taxonomic Composition of Healthy Simmental Calves during Preweaning Period

**Figure 3:**
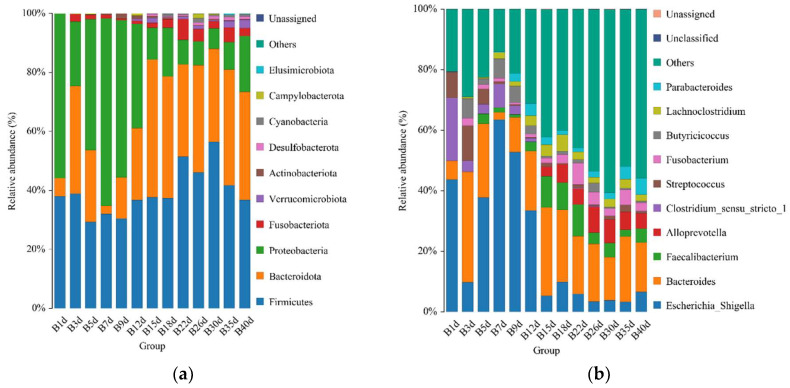

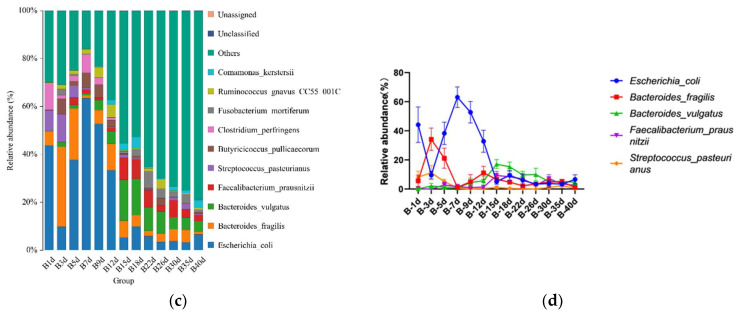
Gut microbial composition of calves at different time points in the healthy group. (**a**) Relative abundance of the top 10 microbial compositions at the phylum level. (**b**) Relative abundance of the top 10 microbial compositions at the genus level. (**c**) Relative abundance of the top 10 microbial compositions at the species level. (**d**) Dynamic changes in the top 5 bacterial species of calves in the healthy group with increasing age.

For healthy Simmental calves, regardless of age, 20 bacterial phyla were identified in their stool samples throughout the experiment. The top 10 bacterial phyla were Firmicutes (39.586 ± 1.773%), Bacteroidota (29.488 ± 1.821%), Proteobacteria (25.959 ± 2.487%), Fusobacteriota (2.538 ± 0.539%), Verrucomicrobiota (0.772 ± 0.204%), Actinobacteriota (0.454 ± 0.098%), Desulfobacterota (0.453 ± 0.072%), Cyanobacteria (0.400 ± 0.124%), Campylobacterota (0.267 ± 0.105%), and Elusimicrobiota (0.062 ± 0.023%) (Figure 3a). Additionally, 328 bacterial genera were identified at the genus level. The 10 predominant bacterial genera were *Escherichia-Shigella* (20.628 ± 2.483%), *Bacteroides* (19.208 ± 1.504%), *Faecalibacterium* (4.374 ± 0.627%), *Alloprevotella* (3.330 ± 0.559%), *Clostridium sensu stricto 1* (2.367 ± 0.639%), *Streptococcus* (2.269 ± 0.520%), *Fusobacterium* (2.538 ± 0.539%), *Butyricicoccus* (2.433 ± 0.429%), *Lachnoclostridium* (2.338 ± 0.253%), and *Parabacteroides* (2.081 ± 0.300%) (Figure 3b). Moreover, 503 bacterial species were identified, with *E.coli* (20.628 ± 2.483%), *B. fragilis* (8.023 ± 1.309%), *B. vulgatus* (6.589 ± 0.830%), *F. prausnitzii* (3.837 ± 0.602%), *S. pasteurianus* (2.103 ± 0.520%), *B. pullicaecorum* (2.308 ± 0.432%), *C. perfringens* (1.717 ± 0.468%), *F. mortiferum* (1.939 ± 0.501%), *R. gnavus_CC55_001C* (1.769 ± 0.355%), and *C. kerstersii* (1.447 ± 0.433%) being the 10 predominant bacterial species (Figure 3c). Furthermore, no significant difference in the relative abundance of the top five bacterial species was detected between adjacent time points (Figure 3d).

### 3.3. Taxonomic Composition of Simmental Calves Infected with Diarrhea during Preweaning Period

**Figure 4:**
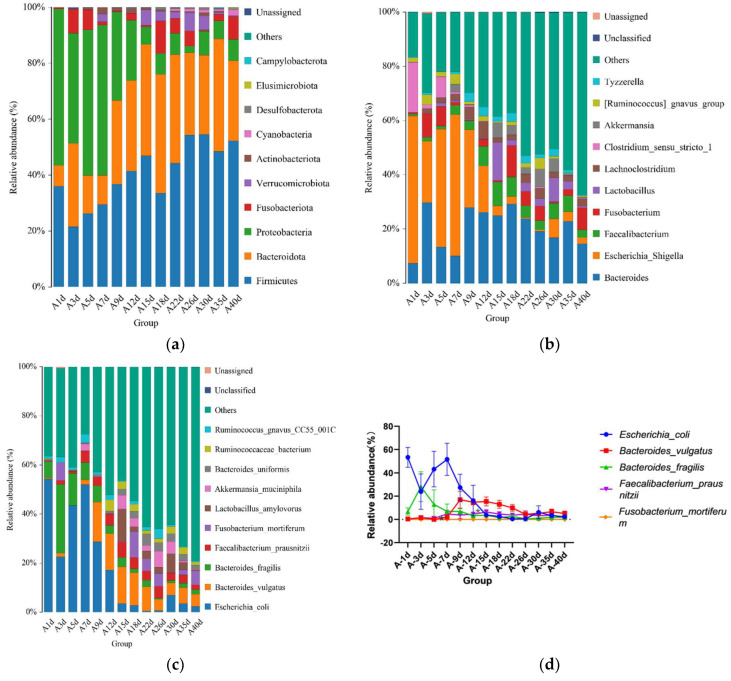
Gut microbial composition of calves at different time points in the diarrheal group. (**a**) Relative abundance of the top 10 microbial compositions at the phylum level. (**b**) Relative abundance of the top 10 microbial compositions at the genus level. (**c**) Relative abundance of the top 10 microbial compositions at the species level. (**d**) Dynamic changes in the top 5 bacterial species of calves in the diarrheal group with increasing age.

For the calves in the diarrheal group, 17 phyla were detected throughout the experiment regardless of age. The top 10 bacterial phyla included Firmicutes (41.492 ± 2.317%), Bacteroidota (29.384 ± 2.341%), Proteobacteria (21.615 ± 3.043%), Fusobacteriota (3.815 ± 1.009%), Verrucomicrobiota (2.170 ± 0.564%), Actinobacteriota (0.722 ± 0.188%), Cyanobacteria (0.480 ± 0.133%), Desulfobacterota (0.292 ± 0.066%), Elusimicrobiota (0.119 ± 0.0019%), and Campylobacterota (0.020 ± 0.016%) (Figure 4a). Additionally, 135 bacterial genera were identified, of which *Bacteroides* (20.756 ± 1.895%) was the most dominant, followed by *Escherichia-Shigella* (17.129 ± 3.057%), *Faecalibacterium* (4.433 ± 0.689%), *Fusobacterium* (3.813 ± 1.099%), *Lactobacillus* (2.971 ± 1.265%), *Lachnoclostridium* (2.383 ± 0.989%), *Clostridium_sensu_stricto_1* (2.654 ± 0.342%), *Akkermansia* (2.164 ± 0.564%), *[Ruminococcus]_gnavus_group* (1.646 ± 0.374%), and *Tyzzerella* (1.751 ± 0.276%) (Figure 4b). A total of 458 bacterial species were identified at the species level. The top 10 annotated species were *E. coli* (17.129 ± 3.057%), *B. vulgatus* (7.695 ± 1.051%), *B. fragilis* (5.308 ± 1.339%), *F. prausnitzii* (3.449 ± 0.536%), *F. mortiferum* (2.598 ± 0.977%), *L. amylovorus* (2.638 ± 1.186%), *A. muciniphila* (2.16 4 ± 0.564%), *B. uniformis* (1.909 ± 0.351%), *R. bacterium* (1.713 ± 0.432%), and *R. gnavus_CC55_001C* (1.451 ± 0.345%) (Figure 4c).

Moreover, significant changes in the relative abundances of the top five bacterial species were observed between adjacent time points. The results showed significant differences in the relative abundance of *B. fragilis* between days 5 (13.000 ± 12.460%) and 7 (6.868 ± 6.322%) (*p* = 0.047), and *F. prausnitzii* exhibited significant differences between days 12 (4.573 ± 1.396%) and 15 (6.377 ± 2.011%) (*p* = 0.046) (Figure 4d). No significant differences were observed in the relative abundances of other bacteria at the species level.

### 3.4. Similarity Analysis of Gut Microbiota of Simmental Calves during Preweaning Period

In our study, ANOSIM analysis was used to conduct pairwise comparisons of gut microbial structures in preweaning calves between the healthy and diarrheal groups at 13 time points. In the healthy group, the gut microbiota exhibited similarity between days 1 and 7 (*r* < 0.5), as well as between days 18 and 40 (*r* < 0.5). However, the microbial structure on day 12 differed between the two age groups (Table 1). In the diarrheal group, similar gut microbial patterns were observed in two age groups, between days 1 and 15 (*r* < 0.5) as well as between days 18 and 35 (*r* < 0.5) (Table 2).

### 3.5. Differential Analysis of Intestinal Microbiota of Simmental Calves between Healthy and Diarrheal Groups

In this study, we conducted a comparative analysis to identify potential differences in bacterial taxonomy between calves in the healthy and diarrheal groups from birth through the stage of diarrheal resolution (1, 3, 5, 7, and 9 days). Notably, on day 1 after birth, significant differences were observed in the relative abundances of three bacterial species between calves in the healthy and diarrheal groups. Specifically, *Limosilactobacillus* (diarrheal group: 0, healthy group: 0.035 ± 0.021%, *p* = 0.046) and *P. mirabilis* (diarrheal group: 0.073 ± 0.071%, healthy group: 0.043 ± 0.01%, *p* = 0.032) exhibited higher relative abundances in the diarrheal group than in the healthy group. Conversely, *L. johnsonii* showed a lower relative abundance in the diarrheal group than in the healthy group (diarrheal group: 0.012 ± 0.010%, healthy group: 0.030 ± 0.019%, *p* = 0.012) (Table 3).

On day 3 following birth, significant differences were observed in the relative abundances of four bacterial species between both groups. Four bacterial species displayed significantly lower relative abundances in the diarrheal group than in the healthy group, including the relative abundance of *F. prausnitzii* (diarrheal group: 0.029 ± 0.023%, healthy group: 1.619 ± 1.461%, *p* = 0.014), *P. russellii* (diarrheal group: 0.023 ± 0.018%, healthy group: 1.330 ± 0.720%, *p* = 0.008), *E. ramosum* (diarrheal group: 0.778 ± 0.416%, healthy group: 2.283 ± 0.611%, *p* = 0.023), and *L. johnsonii* (diarrheal group: 0, healthy group: 1.208 ± 1.113%, *p* = 0.023) (Table 3).

On day 5 after birth, when diarrhea occurred, the relative abundance of three bacterial species showed significantly lower relative abundances in the diarrheal group compared with those in the healthy group: *F. bumbilicata* (diarrheal group: 0, healthy group: 0.269 ± 0.159%, *p* = 0.010), *G. bgenomosp 3* (diarrheal group: 0.057 ± 0.037%, healthy group: 2.764 ± 1.556%, *p* = 0.019), and *C. bpharyngocola* (diarrheal group: 0.309 ± 0.309%, healthy group: 1.376 ± 0.609%, *p* = 0.040) (Table 3).

On day 7 after birth, the relative abundances of five bacterial species were markedly higher in the diarrheal group than in the healthy group, including *S. mitis* (diarrheal group: 0.380 ± 0.380%, healthy group: 0.005 ± 0.008%, *p* = 0.013), *E. ramosum* (diarrheal group: 0.638 ± 0.252%, healthy group: 0.087 ± 0.053%, *p* = 0.019), *P. mirabilis* (diarrheal group: 0.180 ± 0.099%, healthy group: 0.026 ± 0.017%, *p* = 0.019), *A. muciniphila* (diarrheal group: 2.96: ± 2.760%, healthy group: 0.004 ± 0.003%, *p* = 0.040), and *L. amylovorus* (diarrheal group: 0.011 ± 0.004%, healthy group: 0.002 ± 0.001%, *p* = 0.040) (Table 3).

After the recovery phase of calf diarrhea, specifically on day 9 after birth, significant differences were noted in the relative abundance of 10 bacterial species between both groups. Five bacterial species exhibited higher relative abundances in the diarrheal group than in the healthy group: *L. murinus* (diarrheal group: 8.123 ± 7.134%, healthy group: 0.128 ± 0.127%, *p* = 0.010), *F. necrophorum* (diarrheal group: 0.071 ± 0.042%, healthy group: 0.001 ± 0.001%, *p* = 0.012), *A. muciniphila* (diarrheal group: 0.159 ± 0.096%, healthy group: 0.008 ± 0.006%, *p* = 0.020), *B. vulgatus* (diarrheal group: 16.951 ± 7.283%, healthy group: 4.473 ± 2.922%, *p* = 0.020), and *K. pneumoniae* (diarrheal group: 1.384 ± 1.285%, healthy group: 0.003 ± 0.002%, *p* = 0.028). Meanwhile, the relative abundances of five other bacterial species in the diarrheal group were significantly lower than those in the healthy group: *P. russellii* (diarrheal group: 0.032 ± 0.026%, healthy group: 6.153 ± 5.149%, *p* = 0.004) *S. mitis* (diarrheal group: 0.012 ± 0.018%, healthy group: 8.893 ± 0.003%, *p* = 0.012), *P. dorei* (diarrheal group: 0.003 ± 0.003%, healthy group: 0.005 ± 0.005%, *p* = 0.020), *E. coli* (diarrheal group: 27.465 ± 11.439%, healthy group: 52.753 ± 7.410%, *p* = 0.039), and *L. johnsonii* (diarrheal group: 0.262 ± 0.253%, healthy group: 2.409 ± 1.656%, *p* = 0.020) (Table 3).

### 3.6. Prediction of Intestinal Microbial Functions of Simmental Calves

**Figure 5:**
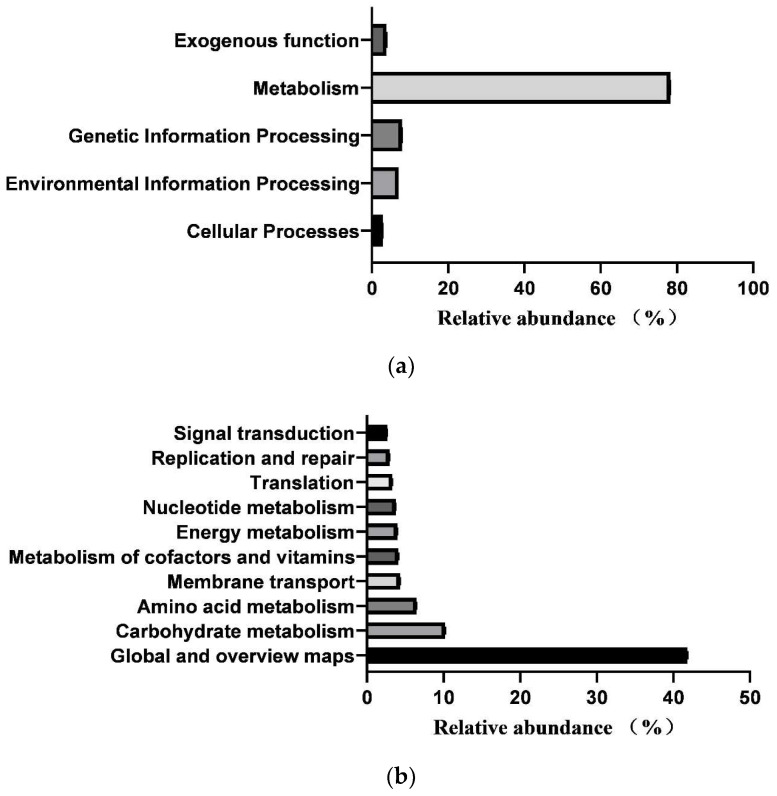

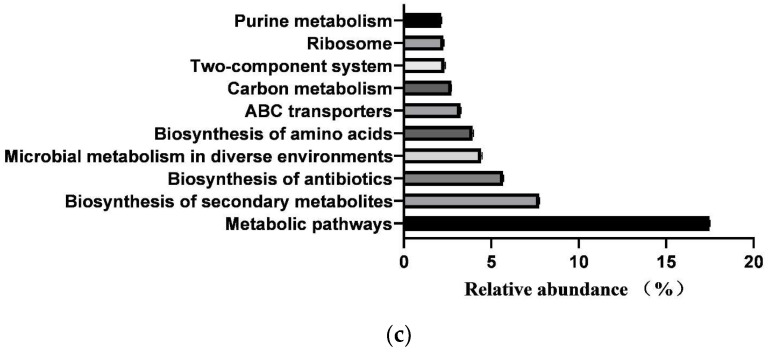
Prediction of intestinal microbial functions in healthy preweaning Simmental calves. (**a**) Top 5 first-level KEGG pathways. (**b**) Top 5 second-level KEGG pathways. (**c**) Top 10 KEGG pathways.

In total, 364 KEGG pathways (metabolic pathways with CPM > 5 in at least 50% of the animals in each treatment group) were predicted from fecal samples of healthy neonatal calves. However, 25 pathways classified as exogenous were subsequently removed, leaving 181 pathways for further analysis (Supplementary File S2). These 181 identified KEGG pathways belonged to four first-level KEGG functions, which were “Cellular Processes” (2.900 ± 0.029%), “Environmental Information Processing” (7.001 ± 0.104%), “Genetic Information Processing” (7.941 ± 0.083%), and “Metabolism” (78.293 ± 0.093%) (Figure 5a). Additionally, 27 secondary-level KEGG functions were identified, with “Global and overview maps” (41.855 ± 0.084%), “Carbohydrate metabolism” (10.202 ± 0.056%), “Amino acid metabolism” (6.479 ± 0.033%), “Membrane transport” (4.313 ± 0.073%), “Metabolism of cofactors and vitamins” (4.136 ± 0.017%), “Energy metabolism” (3.999 ± 0.015%), “Nucleotide metabolism” (3.752 ± 0.025%), “Translation” (3.309 ± 0.044%), “Replication and repair” (2.971 ± 0.029%), and “Signal transduction” (2.649 ± 0.039%) being the top 10 functions (Figure 5b). Furthermore, the top 10 KEGG pathways were “Metabolic pathways” (17.483 ± 0.031%), “Biosynthesis of secondary metabolites” (7.743 ± 0.021%), “Biosynthesis of antibiotics” (5.684 ± 0.023%), “Microbial metabolism in diverse environments” (4.422 ± 0.032%), “Biosynthesis of amino acids” (3.942 ± 0.031%), “ABC transporters” (3.230 ± 0.035%), “Carbon metabolism” (2.719 ± 0.005), “Two-component system” (2.312 ± 0.033%), “Ribosome” (2.274 ± 0.024%), and “Purine metabolism” (2.162 ± 0.011%) (Figure 5c).

### 3.7. Differences in Microbial Functions of Calves between Healthy and Diarrheal Groups

**Figure 6:**
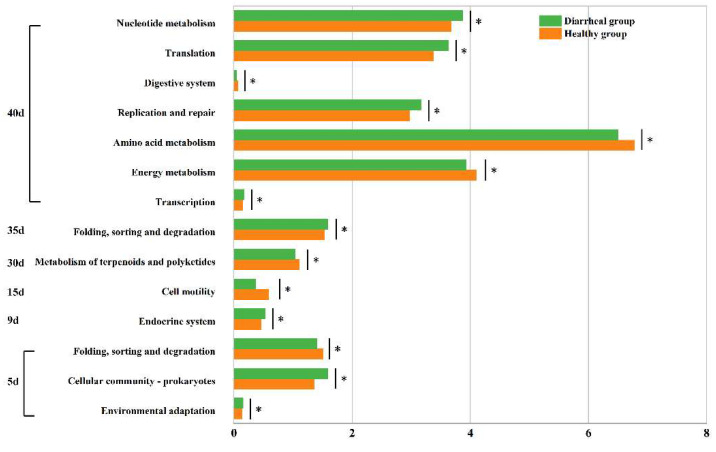
Differential analysis of intestinal microbial functions in Simmental calves between healthy and diarrheal groups before weaning. * represents pairwise comparison with a *p*-value < 0.05.

Second-level KEGG functions were compared between calves in the healthy and diarrheal groups on days 5, 9, 15, 30, 35, and 40. Specifically, on day 5 after birth, the three functions demonstrated significant differences between the two groups. Among these, two functions exhibited significantly higher relative contents in calves in the diarrheal group than in calves in the healthy group, namely “Environmental adaptation” (diarrheal group:0.156 ± 0.010%, healthy group: 0.142 ± 0.009%, *p* = 0.046) and “Cellular community-prokaryotes” (diarrheal group: 1.588 ± 0.162%, healthy group: 1.361 ± 0.124%, *p* = 0.047). Conversely, the relative content of the “Folding, sorting and degradation” function was notably lower in diarrheal group calves compared to the healthy group (diarrheal group: 1.401 ± 0.072%, healthy group: 1.505 ± 0.069%, *p* = 0.048). On day 9 after birth, following diarrheal recovery, a notable difference was observed between the two groups in one function, with the relative content significantly higher in the diarrheal group compared to the healthy group, “Endocrine system” (diarrheal group: 0.527 ± 0.045%, healthy group: 0.460 ± 0.057%, *p* = 0.044). Similarly, on day 15, a significant difference was noted in one function between both groups, namely “Cell motility” (diarrheal group: 0.369 ± 0.153%, healthy group: 0.587 ± 0.177%, *p* = 0.043). Likewise, on day 30, a single function showed a significant disparity between the groups, namely “Metabolism of terpenoids and polyketides” (diarrheal group: 1.034 ± 0.045%, healthy group: 1.104 ± 0.030%, *p* = 0.016). Additionally, on day 35, one function exhibited a significant difference between the two groups: “Folding, sorting and degradation” (diarrheal group: 1.587 ± 0.036%, healthy group: 1.527 ± 0.028%, *p* = 0.012). On day 40, significant differences were observed in seven functions between the two groups. The relative content of four functions was notably higher in diarrheal group calves than the healthy group: “Transcription” (diarrheal group: 0.173 ± 0.011%, healthy group: 0.153 ± 0.009%, *p* = 0.012), “Replication and repair” (diarrheal group: 3.173 ± 0.107%, healthy group: 2.975 ± 0.160%, *p* = 0.025), “Nucleotide metabolism” (diarrheal group: 3.871 ± 0.146%, healthy group: 3.676 ± 0.152%, *p* = 0.046), and “Translation” (diarrheal group: 3.628 ± 0.119%, healthy group: 3.377 ± 0.239%, *p* = 0.036). Conversely, the relative content of three functions was significantly lower in diarrheal group calves than the healthy group: “Energy metabolism” (diarrheal group: 3.927 ± 0.107%, healthy group: 4.104 ± 0.078%, *p* = 0.013), “Amino acid metabolism” (diarrheal group: 6.500 ± 0.150%, healthy group: 6.780 ± 0.203%, *p* = 0.031), and “Digestive system” (diarrheal group: 0.041 ± 0.016%, healthy group: 0.063 ± 0.013%, *p* < 0.05) (Figure 6).

## 4. Discussion

The health of calves is crucial for both farmers, owing to its effect on farm profitability, and consumers, who are increasingly concerned about the welfare and health of farm animals [14]. In neonatal ruminants, nutrient digestion primarily occurs in the intestine because of underdeveloped rumen. The gut microbiota is crucial for host nutrition, absorption, metabolism, immune regulation, and gut health [33]. In this study, 14 preweaning Simmental calves were selected, and their stool samples were subjected to high-throughput sequencing of the V1–V9 region of the 16S rRNA gene. Microbial profiling has revealed diverse and dense microbial colonization in neonatal calves [34]. Throughout the experimental period, α-diversity indices, including Chao1, Simpson, and ACE, showed no significant differences with age in both healthy and diarrheal groups, which may be attributed to individual variation or the limited number of preweaning calves. In contrast to our findings, significant differences were observed in Chao1 and Shannon indices among different age groups of Holstein calves [34]. Such discrepancies may arise from breed differences between Holstein and Simmental calves or variations in the sampling points.

In healthy Simmental calves, analysis of intestinal microbiota changes during the preweaning period using PCoA revealed structural shifts in the gut microbiome. The intestinal microbial structure of calves remained similar from days 1 to 9, with a noticeable transition on day 12, likely linked to intestinal maturation and changes in feed intake. However, feed intake was not measured in this study, highlighting the need for further investigation to explore the correlation between feed intake and intestinal microbial colonization. Additionally, the microbial structure remained similar from days 15 to 40, suggesting that microorganisms tended to mature and stabilize during this period. Our findings align with those of previous studies on Holstein calves, which also exhibited age-related changes, with microbial structures on days 21 and 42 resembling each other but differing from day 7 of the preweaning period [34]. Both studies indicated a gradual maturation process of the intestinal microbiota, although the timing of transition to maturity and stability may vary based on the calf breed and sampling time.

Similar to the results of previous studies on calf fecal intestinal microbiota targeting the V1–V4 hypervariable regions of the 16S rRNA gene through 454 pyrosequencing [35], Firmicutes, Bacteroidetes, and Proteobacteria were the dominant phyla in preweaning Simmental calves, constituting over 90% of the total bacterial composition. Moreover, *E. coli*, *B. fragilis*, and *B. vulgatus* emerged as the dominant species, consistent with studies on dynamic changes in the intestinal microbiota of preweaning Holstein calves [36]. *Escherichia*, as facultative anaerobes, can create a conducive environment for anaerobic bacterial colonization soon after calf birth [37]. Therefore, the high abundance of *Escherichia* is closely related to intestinal oxygen consumption. Additionally, *Bacteroides* metabolize nutrients to produce acetic acid, which serves as a substrate for Butyricoccus and Megamonas to produce butyric and propionic acids [38]. Butyrate is crucial for intestinal epithelial cells as an energy source [39] and for inhibiting proinflammatory cytokine signaling pathways [40]. Substantial colonization by *E. coli*, *B. fragilis*, and *B. vulgatus* during early life plays an important role in gut health, potentially contributing to the maturation and functionality of intestinal processes.

During days 5–7, coinciding with the onset of diarrhea, there was a significant change in the relative abundance of *B. vulgatus* in calves in the diarrheal group [41]. The reduction in the relative abundance of *B. vulgatus* may potentially influence the growth and health of preweaning calves affected by diarrhea. Previous studies have suggested that oral administration of *F. prausnitzii* significantly reduces the incidence of severe diarrhea and mortality in lactating Holstein calves while promoting growth and intestinal health [42]. In our study, the relative abundance of *F. prausnitzii* in the gut microbiota of calves in the diarrheal group was higher on day 15 than on day 12, indicating an increase after the calves recovered from diarrhea. This finding aligns with previous research highlighting the role of *F. prausnitzii* in neonatal calf intestinal health. Comparative analysis revealed significant differences in the relative abundance of intestinal microbiota between calves in the healthy and diarrheal groups at the corresponding sample collection points. Studies on preweaning Holstein calves have shown that milk supplemented with *Lactobacillus reuteri L81* and *Lactobacillus johnsonii L29* enhances growth performance, immunity, and antioxidant capacity, while reducing the incidence of diarrhea [43]. Interestingly, a higher relative abundance of *L. johnsonii* was detected in the intestines of Simmental calves in the healthy group on days 1, 3, and 9, suggesting a potential probiotic effect of *L. johnsonii* closely related to the absence of diarrhea in these calves. The oral administration of *F. prausnitzii* is closely associated with a reduced rate of calf diarrhea [44]. In our study, the relative abundance of *F. prausnitzii* was significantly higher in the intestines of calves in the healthy group on day 3 after birth, possibly contributing to the absence of diarrhea in this group. Meanwhile, *Limosilactobacillus* supplementation has been shown to alleviate the symptoms of *ETEC K88*-induced diarrhea in piglets by modulating macrophage phenotypes [45]. Therefore, the higher relative abundance of *Limosilactobacillus* observed on day 1 in neonatal Simmental calves could potentially contribute to the prevention of calf diarrhea. Therefore, *L. johnsonii*, *F. prausnitzii*, and *Limosilactobacillus* may play significant roles in preventing diarrhea in Simmental calves and could be developed as potential probiotic bacteria. Conversely, *E. coli* is a potentially pathogenic bacterium that causes neonatal calf diarrhea [46]. On day 9, shortly after recovery from diarrhea, the relative abundance of *E. coli* in the diarrheal group was significantly higher than in the healthy group, suggesting a heightened risk of recurrent diarrhea in calves in the diarrheal group.

In contrast to the predicted main intestinal functions of Holstein calves [34], the core functions identified in preweaning Simmental calves not only included functions related to nutrient metabolism but also encompassed “Biosynthesis of antibiotics”, which may be linked to breed-specific productive properties. This finding suggests that the gut microbes of preweaning Simmental calves may tend to promote intestinal health through antibiotic production. Furthermore, differences in intestinal microbial functions between calves in the healthy and diarrheal groups were examined. A significantly higher relative abundance of “Environmental adaptation” function was observed on day 5 in the healthy group, indicating that the intestinal microbiota of healthy calves exhibited stronger adaptability to the intestinal environment and maintained better homeostasis [47]. By day 40, the relative abundances of “energy metabolism” and “amino acid metabolism” functions in the healthy group surpassed those in the diarrheal group. These functions are crucial in the hindgut of preweaning calves and provide essential energy and nutrients to the host [48]. Therefore, our findings suggest that the gut microbiome of calves in the healthy group may contribute more effectively to providing energy and nutrients to the host than calves in the diarrheal group.

In future animal husbandry practices, veterinarians, veterinary technicians, and farmers should receive training in veterinary knowledge to understand the critical importance of early intestinal health in young livestock [49]. The development and application of probiotics are crucial for promoting the growth and development of young calves. Furthermore, educating modern students about 16S rRNA gene V1–V9 sequencing technology and its practical applications in classrooms will contribute to cultivating knowledgeable students and skilled veterinarians [50].

## 5. Conclusions

This study investigated the dynamic changes in the intestinal microbiota of preweaning Simmental calves and predicted the functions of their gut microbiota. *E. coli*, *B. fragilis*, and *B. vulgatus* emerged as dominant bacterial species. Major intestinal functions included “Biosynthesis of secondary metabolites”, “Biosynthesis of antibiotics”, “Microbial metabolism in diverse environments”, and “Biosynthesis of amino acids”. Moreover, pronounced dynamic changes in the intestinal microbiota of calves in both healthy and diarrheal groups were observed with increasing age. Although further understanding is needed on the interactions between miRNA/mRNA–TLR–microbiome, our analysis provides insights into their role as modulators in communication between biological processes and metabolic pathways, which are crucial for establishing innate and adaptive immunity during the transition from calves to ruminants. Furthermore, significantly higher relative abundances of *L. johnsonii*, *F. prausnitzii*, and *Limosilactobacillus* were detected in the gut of the calves in the healthy group, which may be closely related to the absence of diarrhea. Our study offers valuable insights into the prevention of diarrhea and development of probiotics for Simmental calves.

## Figures and Tables

**Table 1:** ANOSIM pairwise comparison matrix of fecal microbiota of calves in the healthy group (*n* = 8).

Healthy Group	1 d	3 d	5 d	7 d	9 d	12 d	15 d	18 d	22 d	26 d	30 d	35 d	40 d
1 d	0												
3 d	*r* = 0.398 *	0											
5 d	*r* = 0.402 *	***r* = −0.001**	0										
7 d	*r* = 0.340 *	*r* = 0.363 *	*r* = 0.328 *	0									
9 d	*r* = 0.718 *	*r* = 0.544 *	*r* = 0.491 *	***r* = −0.021**	0								
12 d	*r* = 0.861 *	*r* = 0.789 *	*r* = 0.667 *	*r* = 0.199 *	*r* = 0.137 *	0							
15 d	*r* = 0.889 *	*r* = 1 *	*r* = 0.988 *	*r* = 0.871 *	*r* = 0.954 *	*r* = 0.646 *	0						
18 d	*r* = 1 *	*r* = 1 *	*r* = 0.996 *	*r* = 0.947 *	*r* = 0.992 *	*r* = 0.845 *	***r* = 0.027**	0					
22 d	*r* = 1 *	*r* = 0.985 *	*r* = 0.978 *	*r* = 0.925 *	*r* = 0.951 *	*r* = 0.801 *	*r* = 0.388 *	***r* = 0.122**	0				
26 d	*r* = 0.985 *	*r* = 0.961 *	*r* = 0.962 *	*r* = 0.900 *	*r* = 0.934 *	*r* = 0.735 *	*r* = 0.314 *	***r* = 0.068**	***r* = −0.109**	0			
30 d	*r* = 1 *	*r* = 1 *	*r* = 0.996 *	*r* = 0.965 *	*r* = 0.996 *	*r* = 0.907 *	*r* = 0.525 *	*r* = 0.213 *	***r* = −0.034**	*r* = −0.634	0		
35 d	*r* = 1 *	*r* = 1 *	*r* = 0.999 *	*r* = 0.996 *	*r* = 1 *	*r* = 0.969 *	*r* = 0.824 *	*r* = 0.422 *	*r* = 0.181 *	***r* = 0.007**	***r* = −0.047**	0	
40 d	*r* = 1 *	*r* = 0.998 *	*r* = 0.991 *	*r* = 0.980 *	*r* = 0.996 *	*r* = 0.906 *	*r* = 0.611 *	*r* = 0.413 *	*r* = 0.252 *	***r* = 0.014**	***r* = 0.091**	***r* = 0.064**	0

* represents pairwise comparison with a *p*-value < 0.05. Values with *r* < 0.5 are bolded. When *p*-value < 0.05, fecal microbiotas between two age groups were considered completely different at *r*-value > 0.75; different at 0.5 < *r*-value < 0.75; tended to be different data 0.3 < *r*-value < 0.5; not different at *r*-value < 0.3.

**Table 2:** ANOSIM pairwise comparison matrix of fecal microbiota of calves in the diarrheal group (*n* = 6).

Diarrhea Group	1 d	3 d	5 d	7 d	9 d	12 d	15 d	18 d	22 d	26 d	30 d	35 d	40 d
1 d	0												
3 d	***r* = 0.017**	0											
5 d	*r* = 0.362 *	***r* = 0.125**	0										
7 d	*r* = 0.533 *	*r* = 0.409 *	***r* = 0.022**	0									
9 d	*r* = 0.635 *	*r* = 0.619 *	***r* = 0.024**	***r* = −0.192**	0								
12 d	*r* = 0.861 *	*r* = 0.833 *	*r* = 0.709 *	*r* = 0.347 *	***r* = 0.128**	0							
15 d	*r* = 0.938 *	*r* = 1 *	*r* = 0.924 *	*r* = 0.728 *	*r* = 0.425 *	***r* = −0.099**	0						
18 d	*r* = 0.942 *	*r* = 0.976 *	*r* = 0.887 *	*r* = 0.728 *	*r* = 0.474 *	***r* = 0.039**	***r* = −0.110**	0					
22 d	*r* = 0.983 *	*r* = 0.996 *	*r* = 0.979 *	*r* = 0.867 *	*r* = 0.648 *	***r* = 0.109**	***r* = 0.051**	***r* = −0.011**	0				
26 d	*r* = 0.935 *	*r* = 0.976 *	*r* = 0.864 *	*r* = 0.715 *	*r* = 0.611 *	***r* = 0.094**	***r* = 0.132**	***r* = −0.057**	***r* = −0.185**	0			
30 d	*r* = 0.972 *	*r* = 0.968 *	*r* = 0.949 *	*r* = 0.888 *	*r* = 0.709 *	*r* = 0.240 *	***r* = 0.174**	***r* = −0.002**	***r* = −0.067**	***r* = −0.204**	0		
35 d	*r* = 0.998 *	*r* = 1 *	*r* = 0.997 *	*r* = 0.992 *	*r* = 0.898 *	*r* = 0.583 *	*r* = 0.462 *	*r* = 0.302 *	***r* = −0.019**	***r* = −0.093**	***r* = −0.004**	0	
40 d	*r* = 0.959 *	*r* = 0.968 *	*r* = 0.947 *	*r* = 0.912 *	*r* = 0.863 *	*r* = 0.561 *	*r* = 0.581 *	*r* = 0.435 *	***r* = 0.146**	***r* = 0.056**	***r* = 0.105**	***r* = −0.098**	0

* represents pairwise comparison with a *p*-value < 0.05. Values with *r* < 0.5 are bolded. When *p*-value < 0.05, fecal microbiotas between two age groups were considered completely different at *r*-value > 0.75; different at 0.5 < *r*-value < 0.75; tended to be different data 0.3 < *r*-value < 0.5; not different at *r*-value < 0.3.

**Table 3:** Relative abundance comparison of intestinal microbiota between calves in the healthy and diarrheal groups at different time points.

Day	Bacteria	Group		*p*-Value
		A	B	
1 d	*L. johnsonii*	0.012 ± 0.010%	0.030 ± 0.019%	0.012
	*Limosilactobacillus*	0	0.035 ± 0.021%	0.046
	*P. mirabilis*	0.073 ± 0.071%	0.043 ± 0.017%	0.032
3 d	*P. russellii*	0.023 ± 0.018%	1.330 ± 0.720%	0.008
	*F. prausnitzii*	0.029 ± 0.023%	1.619 ± 1.461%	0.014
	*E. ramosum*	0.778 ± 0.416%	2.283 ± 0.611%	0.023
	*L. johnsonii*	0.002 ± 0.002%	1.208 ± 1.113%	0.023
5 d	*F. umbilicata*	0	0.269 ± 0.159%	0.010
	*G. genomosp._3*	0.057 ± 0.037%	2.764 ± 1.556%	0.019
	*C. pharyngocola*	0.309 ± 0.309%	1.376 ± 0.609%	0.040
7 d	*S. mitis*	0.380 ± 0.380%	0.005 ± 0.008%	0.013
	*E. ramosum*	0.638 ± 0.252%	0.087 ± 0.053%	0.019
	*P. mirabilis*	0.180 ± 0.099%	0.026 ± 0.017%	0.019
	*A. muciniphila*	2.967 ± 2.760%	0.004 ± 0.003%	0.040
	*L. amylovorus*	0.011 ± 0.004%	0.002 ± 0.001%	0.040
9 d	*P. russellii*	0.032 ± 0.026%	6.153 ± 5.149%	0.004
	*L. murinus*	8.123 ± 7.134%	0.128 ± 0.127%	0.010
	*F. necrophorum*	0.071 ± 0.042%	0.001 ± 0.001%	0.012
	*S. mitis*	0.012 ± 0.018%	8.893 ± 0.003%	0.012
	*A. muciniphila*	0.159 ± 0.096%	0.008 ± 0.006%	0.020
	*B. vulgatus*	16.951 ± 7.283%	4.473 ± 2.922%	0.020
	*L. johnsonii*	0.262 ± 0.253%	2.409 ± 1.656%	0.020
	*P. dorei*	0.003 ± 0.003%	0.005 ± 0.005%	0.020
	*K. pneumoniae*	1.384 ± 1.285%	0.003 ± 0.002%	0.028
	*E. coli*	27.465 ± 11.439%	52.753 ± 7.410%	0.039

## Data Availability

The data that support the findings of this study are available from the corresponding authors upon reasonable request.

## Supplementary Materials

The following supporting information can be downloaded at: https://www.mdpi.com/article/10.3390/biology13070520/s1, Supplementary File S1 is Calf Health Monitoring Scoring Criteria and Calf fecal scoring criteria, Supplementary File S2 is Circular Consensus Sequencing, OTU_Num and Seqs_Num, Supplementary File S3 is Alpha Diversity Index statistics.
