# Supplementary material for: Dynamic Analysis of Stool Microbiota of Simmental Calves and Effects of Diarrhea on Their Gut Microbiota
Source: Biology (Basel). 2024 Jul 13;13(7):520. doi: 10.3390/biology13070520 (PMC11273684; doi:10.3390/biology13070520)
Supplement: Supplementary file 1 [file biology-13-00520-s001.zip › Supplementary File S1.pdf]

Table 1 Calf Health Monitoring Scoring Criteria

Clinical parameters	score			
	0	1	2	3
nasal secretion	Normal plasma secretion	Small amount of unilateral secretion	Moderate bilateral discharge	Profuse bilateral mucopurulent discharge
eye secretion	normalcy	Small amount of eye discharge	Moderate bilateral discharge	Heavy eye discharge
cough	normalcy	Inducing a single cough	Induces recurrent or occasional spontaneous coughing	Recurrent spontaneous cough
corditis	normalcy	Slightly swollen, not hot or painful	Slightly enlarged, slightly painful or moist	Increased fever, pain or foul-smelling discharge
rectal temperature	37.8–38.2℃	38.3–38.8℃	38.9–39.4℃	>39.4℃
dehydration	normalcy	Skin swelling returns to normal within 2s	Skin swelling returns to normal in 2s to 4s	Skin swelling returns to normal within 4s
overall state	Rising, lively and energetic, bright and alert, strong sucking power	Doesn't get up or look slightly sluggish or depressed, eyes not sunken, sucks well	Won't get up, looks sluggish and frustrated, eyes slightly sunken, sucks well	Won't even lift his head, looks very sullen and depressed, mildly depressed, bedraggled sternum, moderately sunken eyes and sticky mucous membranes, sucks poorly

Table 2 Calf fecal scoring criteria

score	0	1	2	3
fecal state	normal feces	Semi-formed loose feces	loose, gruel-like stool	watery feces
