# Supplementary figures and images for: Dynamic Analysis of Stool Microbiota of Simmental Calves and Effects of Diarrhea on Their Gut Microbiota
Source: Biology (Basel). 2024 Jul 13;13(7):520. doi: 10.3390/biology13070520 (PMC11273684; doi:10.3390/biology13070520)

## Slide 1
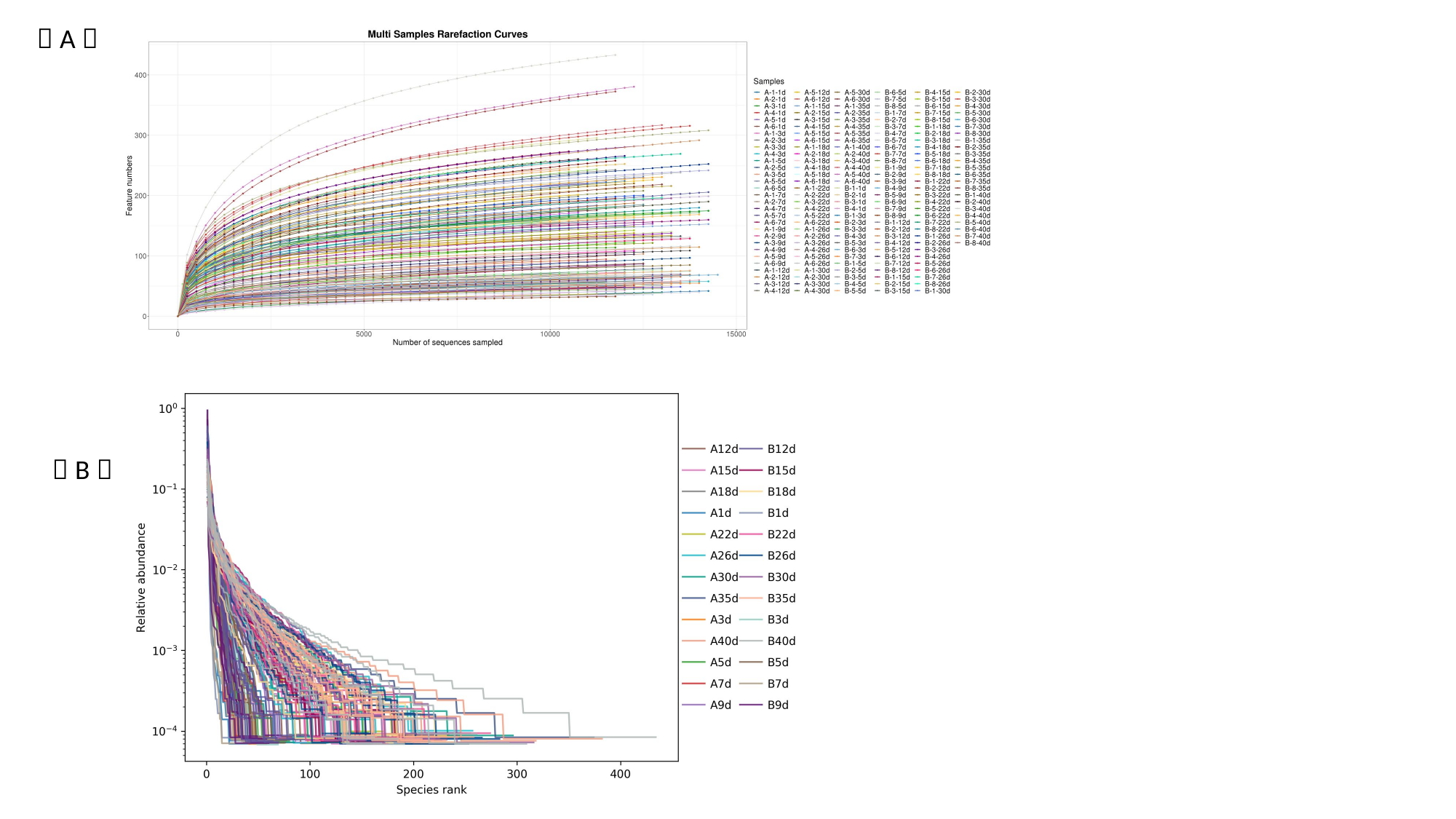

（A）
（B）

## Slide 2
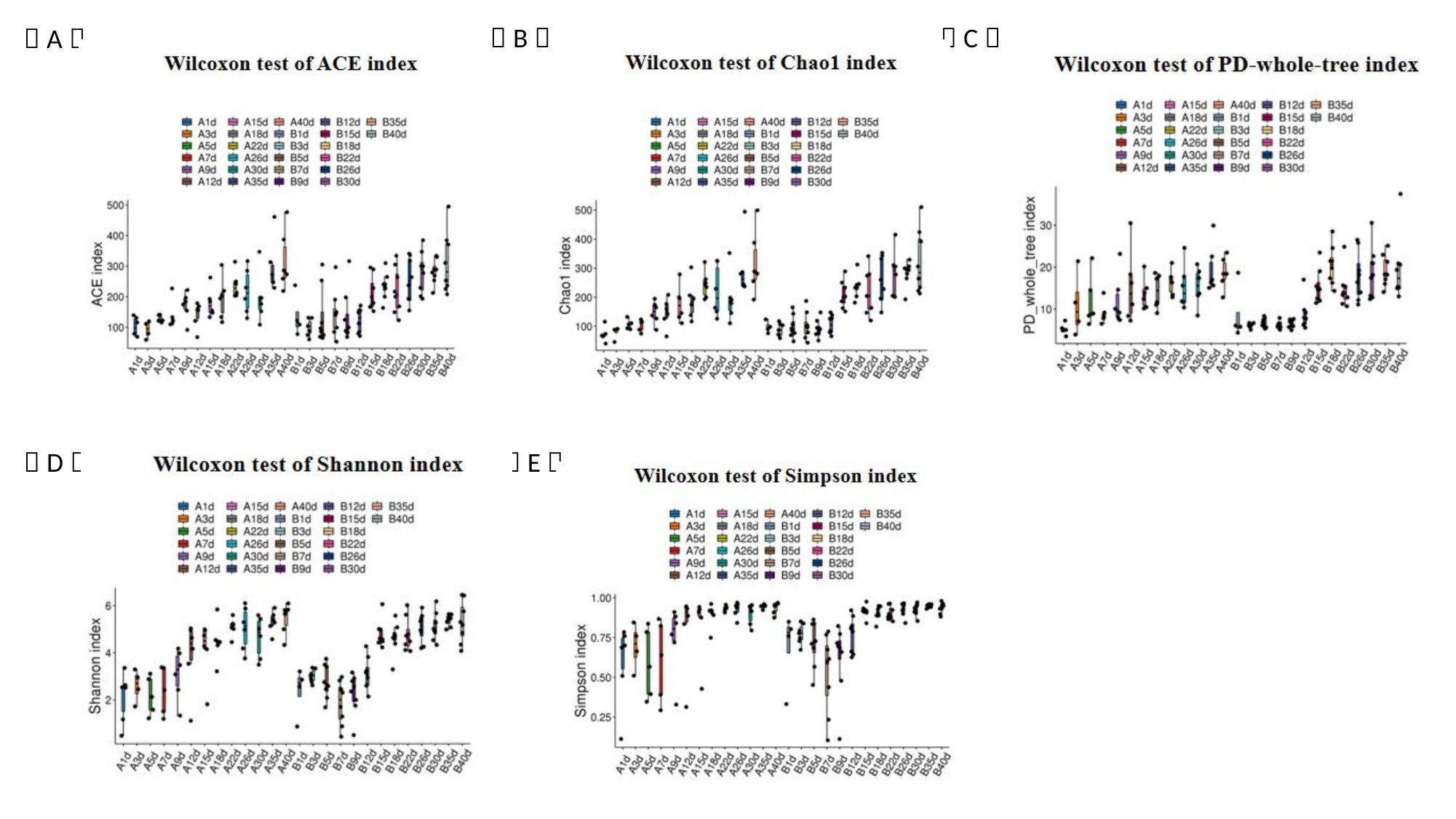

（B）
（C）
（A）
（D）
（E）

Supplement: Supplementary file 1 [file biology-13-00520-s001.zip › Supplementary File S3.pptx]
